# Broken Sleep, Broken Memory? Neurocognitive Consequences of Sleep fragmentation in Older Black Americans

**DOI:** 10.1002/alz70861_108956

**Published:** 2025-12-23

**Authors:** Osswaah Fariduddin, Maria F. Clark, Albert Nyarko, Elizabeth Luth, Darlingtina Esiaka

**Affiliations:** ^1^ University of Kenctucky College of Medicine, Lexington, KY USA; ^2^ University of Kentucky College of Medicine, Lexington, KY USA; ^3^ University of Kentucky, Lexington, KY USA; ^4^ Robert Wood Johnson Medical School, Department of Family Medicine and Community Health, New Brunswick, NJ USA; ^5^ University of Kentucky Sanders‐Brown Center on Aging, Lexington, KY USA

## Abstract

**Background:**

Sleep fragmentation, particularly wake after sleep onset (WASO), is associated with cognitive decline. However, limited research has examined how this relationship manifests in older Black Americans, a population disproportionately affected by brain health disparities and underrepresented in sleep and cognitive research.

**Objective:**

To examine the association between objectively measured WASO and visual‐spatial working memory (VSWM) performance—a sensitive early marker of cognitive impairment—among older Black Americans.

**Methods:**

As part of an ongoing study examining the link between neighborhood factors, sleep and Alzheimer's risk, older Black Americans (*N* =30, Mean age = 53.03) completed three nights of at‐home sleep monitoring using a validated wearable device. VSWM was assessed using standardized cognitive tasks. Pearson correlation was used to examine associations between average WASO and VSWM performance.

**Results:**

Higher levels of WASO were significantly associated with poorer VSWM performance (r = –.386, *p* = .035). This finding suggests that greater sleep fragmentation may be linked to reduced cognitive efficiency in domains dependent on executive functioning, attentional control, and spatial organization.

**Conclusion:**

Sleep continuity, as indicated by lower WASO, appears to be an important factor in preserving cognitive function and reducing the risk for Alzheimer’s disease and related dementias (ADRD). These findings underscore the need for targeted interventions addressing sleep fragmentation to support healthy cognitive aging in this population. Addressing sleep continuity may serve as a modifiable pathway to promote cognitive resilience and mitigate ADRD disparities among Black Americans. Further research is warranted to explore causal mechanisms and potential moderating factors.

